# How aging shapes our sense of agency

**DOI:** 10.3758/s13423-023-02449-1

**Published:** 2024-01-19

**Authors:** Marika Mariano, Nicole Kuster, Matilde Tartufoli, Laura Zapparoli

**Affiliations:** 1 Psychology Department and NeuroMi—Milan Centre for Neuroscience, University of Milano-Bicocca, Milan, Italy; 2 IRCCS Orthopedic Institute Galeazzi, Milan, Italy

**Keywords:** Sense of agency, Aging, Self-awareness, Intentional binding, Judgment of agency

## Abstract

The sense of agency refers to the feeling of controlling one’s actions and their effects on the external environment. Here, we tested how the physiological process of aging affects the agency experience by taking advantage of a validated ecological experimental paradigm and exploring the different dimensions of agency. We tested 60 young and older adults during active and passive movements, causing, after a variable time delay, an external sensorial event. We collected overt agency judgments (i.e., explicit agency dimension), and we measured the perceived compression of the time interval between the active/passive movements and outcomes (to quantify the intentional binding phenomenon, an implicit index of agency). Our results indicate that the sense of agency significantly changes across the adult life span, with older participants exhibiting a reduced sense of agency, both at the explicit and implicit level. Crucially, the temporal dimension of the action outcome did not affect their agency experience. We suggest that elderly adults are more reliant on internal predictions, making them less sensitive to cognitive biases and external manipulations. We discuss these results in the domain of neurocognitive models of motor control, with reference to how aging affects the weighting process of predictive and sensory signals for efficient sensorimotor integration.

The world is experiencing an unprecedented increase in the older population, which is projected to continue for decades to come. Although age-related cognitive decline is commonly observed in multiple domains, only a few studies investigated the impact of physiological aging on cognitive-motor tasks (review in Zapparoli et al., 2022). Yet motor awareness, and in particular, the feeling of actively controlling our voluntary movements (the so-called sense of agency), can be considered a well-suited opportunity to investigate complex mechanisms of functional aging since it relies on an efficient interaction between motor, sensory, and predictive processes.

## The sense of agency: Dimensions, measures, and cognitive models

The term “sense of agency” refers to the experience that accompanies the performance of a specific motor act (Haggard, 2017). Previous researchers have made a clear distinction between two aspects of agency: *judgment of agency* and *feeling of agency* (Synofzik et al., 2008). The first involves consciously evaluating oneself as the agent of an action and its consequences, relying on metacognitive processes. It is mainly measured by using self-report questionnaires.

In contrast, the feeling of agency is a more basic sensation of being the agent of an action, relying on sensorimotor cues and contingencies that are potentially integrated into motor planning processes. It is primarily based on the automatic comparison between predicted and actual outcomes of one’s actions. This feeling of agency can be measured only indirectly, for example, through perceptual estimates of the phenomenology of actions and their sensory consequences. For example, the magnitude of a phenomenon called intentional binding can be used as an indirect estimate of the implicit sense of agency (for a review, see Moore & Obhi, 2012). When a voluntary movement generates an external outcome, people perceive an illusory temporal compression between the movement and the generated outcome. Importantly, this temporal binding seems to reliably occur in situations where the participant is the agent, and to be reduced in the case of outcomes generated by an external event or an externally triggered action, like a TMS-determined muscle contraction (Haggard et al., 2002).

Various theoretical neurocognitive frameworks addressed the agency experience. The comparator model suggests that action control relies on internal models through a series of comparators, whereby a sense of self-agency arises when predictive motor signals match the actual sensory effect of one’s action (Frith et al., 2000).

According to the apparent mental causation theory, the experience of agency is essentially a retrospective inferential and logical reconstruction of events and their probable causes (Wegner, 2003; Wegner & Wheatley, 1999).

According to the comparator model, internal sensorimotor processes are crucial in generating the sense of agency, whereas the apparent mental causation theory emphasizes the importance of external stimuli in causal inference processes. The cue integration theory combines these two models by suggesting that the sense of agency arises from a weighted integration of internal and external cues, depending on the dimension of agency. More specifically, the feeling of agency is influenced by internal predictive cues and subsequently integrated with external cues, leading to the formation of an explicit judgment of agency (Moore et al., 2009; Synofzik et al., 2008).

## Aging and sense of agency

An essential aspect of motor skills is the accurate integration of sensory and motor signals, where incoming information from peripheral sensors is combined with predictive motor signals generated by internal models of movement.

Physiological aging is commonly associated with a decrease in sensory sensitivity accompanied by an increase in sensory noise (Boisgontier & Nougier, 2013; Lafargue et al., 2013). Considering the significance of sensory cues in shaping the sense of agency, it is reasonable to hypothesize that the experience of agency may be somehow influenced by physiological aging.

The few available studies addressing this issue seem in line with this hypothesis, showing a reduced agency experience in older adults (Cioffi et al., 2017; Metcalfe et al., 2010). Metcalfe et al. (2010) also observed that older adults were less sensitive to external manipulations, such as the insertion of temporal or spatial distortion between their actions and the moving of a cursor on the screen. Indeed, their agency judgments did not significantly change when a spatial/temporal delay was artificially inserted between their movements and the movement of the cursor.

This evidence has been further explored by Cioffi and colleagues (Cioffi et al., 2017), demonstrating that older adults are not influenced by external situational cues when judging their agency experience. The authors discussed this finding hypothesizing that elderly individuals can count on more precise and accurate interoceptive and proprioceptive information, thus leading to an increased reliance on internal cues.

Yet it is worth mentioning that these studies were based on somewhat artificial experimental paradigms. In one case, participants moved a cursor on a screen during a video game-like task, which could have been perceived as quite challenging in the case of older adults. In the second study, subjects rated their agency experienced over a nonexecuted action.

Moreover, both these studies focused on the metacognitive and conceptual dimension of the judgment of agency, leaving totally unexplored the lower-level sensorimotor dimension of the feeling of agency.

## Aim of the study and expected results

In this experiment, we want to investigate how aging affects the agency experienced over ecological actions followed by specific external effects. To this aim, we tested young and older adults using a validated experimental paradigm that allows the assessment of the different dimensions of agency.

In a previous experiment, we observed that the perceived sense of agency was significantly stronger when the action outcomes were presented immediately after the execution of the movement, highlighting the crucial role of the temporal dimension for the arising of the sense of agency (Zapparoli, Seghezzi, Zirone et al., 2020b).

Here, we specifically tested whether (i) this temporal effect is replicable in an independent sample of young participants; (ii) such temporal modulation is also measurable in older adults; (iii) age-related changes in the agency experience may be mediated by the agency dimension; (iv) and how these changes can be interpreted in the light of the cognitive models of sense of agency.

We do have some hypotheses in mind, starting from the consideration that the agency experience should rely on the optimal integration between internal sensorimotor predictions and external sensory stimuli (Synofzik et al., 2008). We can anticipate that the presence of noisy sensory information commonly associated with aging would result in a diminished ability to distinguish between self-generated and externally-caused events. Consequently, older participants may experience a decreased sense of agency over their voluntary actions. This decline may also be linked to a weakened modulation of the temporal relationship between actions and their outcomes.

Alternatively, the experience accumulation over the course of one’s life may contribute to an enhanced precision of sensorimotor predictions: older participants may therefore leverage more accurate internal predictive models, leading to a normal sense of agency in the later stages of life. Certainly, it would be interesting to investigate which of these alternative hypotheses is applicable to which dimension of agency.

## Materials and methods

### Participants

We recruited 30 young adult subjects (mean age: 22±1.8 years; mean education level 14.4±1.5 years; male/female ratio: 11/19) and 30 older participants (mean age: 59.5±4.9 years; mean education level 14.4±3.1 years; male/female ratio: 12/18) with no history of neurological or psychiatric illness.

They were all right-handed, as assessed by the Edinburgh Handedness Inventory (Oldfield, 1971). The local Ethics Committee approved the study protocol (protocol RM-2023-644) and informed written consent was obtained from all subjects according to the Helsinki Declaration (1964). All subjects took part in the study after the nature of the procedure had been fully explained and informed consent had been obtained. To exclude subjects with cognitive deficits, we administered the Mini-Mental State Examination (Folstein et al., 1975) to each participant. None of the subjects had pathological scores. Moreover, participants did not have visual problems (i.e., color blindness) that could influence their performance during the task.

### Experimental task

**Fig. 1:**
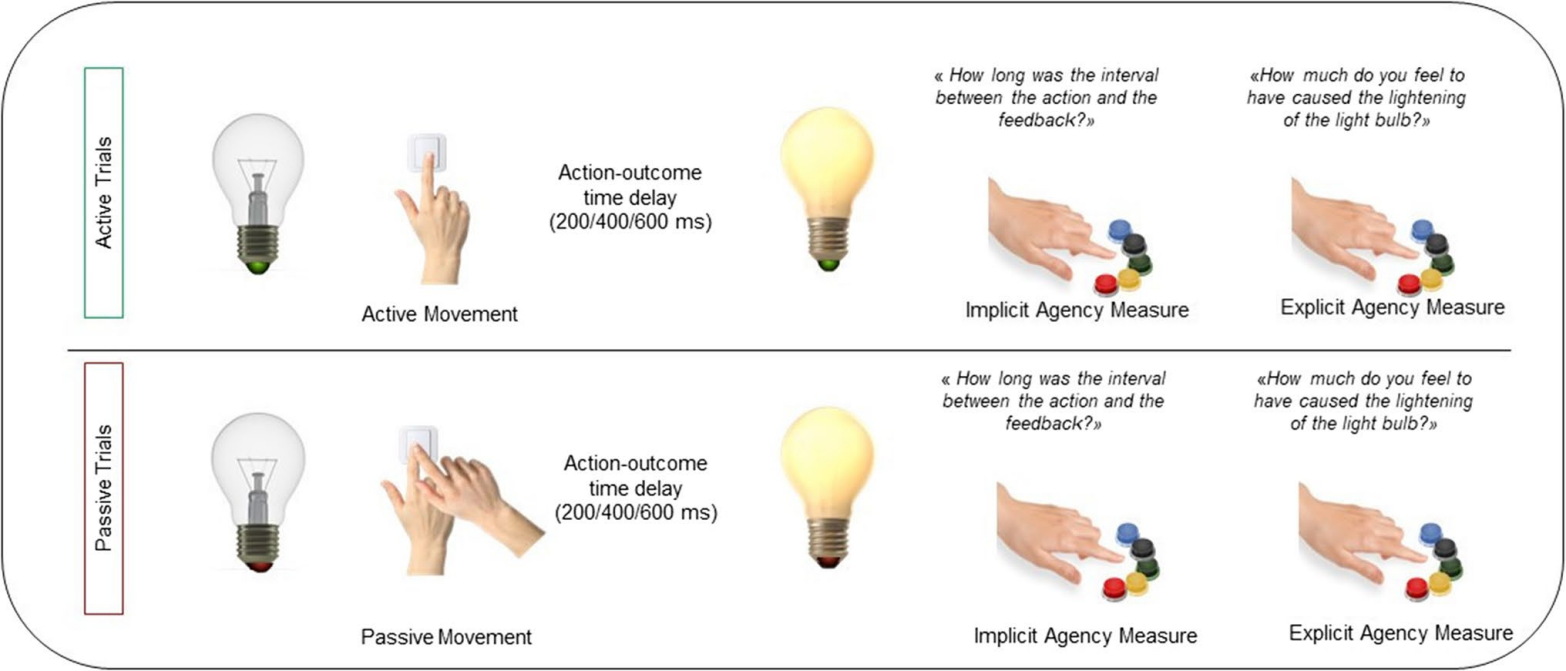
Illustration of an experimental trial (for both active and passive conditions). During active trials, participants pressed a button with their right index finger at their own time after the presentation of the cue. In passive conditions, participants were instructed to stay still while an experimenter pressed their finger to press the button with their right index finger at their own time after the presentation of the cue. In both conditions, the button press caused an action-consequence: the lightening of the light bulb. The consequence was presented after a variable delay of 200, 400, or 600 ms. Participants then judged the perceived time interval between their button press and the lightening of the light bulb (to measure the intentional binding phenomenon, an implicit measure of agency) and how much they felt to have caused, with their action, the lightening of the light bulb (judgment of agency, explicit agency dimension). (Color figure online)

Participants executed a validated temporal judgment task (Seghezzi & Zapparoli, 2020; Zapparoli, Seghezzi, Devoto et al., 2020a; Zapparoli, Seghezzi, Zirone et al., 2020b) with active and passive trials (see Fig. 1). During active trials, the picture of a turned-off light bulb with a green base was shown. Participants were instructed to turn the light bulb on by pressing a button with their right index finger. After the button press, the light bulb went on with a variable delay of 200, 400, or 600 ms. Participants were then asked to rate the perceived time interval between their button press and the lighting of the light bulb. The judgment was reported by means of a visual analog scale at which they responded using a five-key response keypad placed under their left hand. They used their fingers, starting from the thumb to the pinkie finger, and so on, to select one of five possible response options: 1 ms, 200 ms, 400 ms, 600 ms, and 800 ms. The lowest and the highest response options were included to make it possible for the participants to underestimate and overestimate each presented temporal interval.

Moreover, participants were asked how much they felt to have produced, with their action, the lighting of the light bulb (judgment of agency, explicit agency dimension). Again, participants used their left fingers, starting from the thumb (1 = *not at all produced by me*) to the pinkie finger (5 = *completely produced by me*). Both scales remained visible for 5 seconds.

In passive trials, the base of the light bulb was red. Subjects were instructed to stay still while an experimenter pressed their right index finger to produce a passive movement. The passive movement turned the light bulb on. Participants were then asked to judge the action–outcome delay in the same way as for active trials and the perceived contribution over the lighting of the light bulb.

As in our previous works (Seghezzi & Zapparoli, 2020; Zapparoli, Seghezzi, Devoto et al., 2020a; Zapparoli, Seghezzi, Zirone et al., 2020b), we administered 60 trials equally distributed between active and passive trials, with 10 trials for each of the three action–outcome delays. Before the experiment, participants practiced with the task. First, we showed them the different possible time delays between the pressing of the button and the lighting of the lightbulb (they pressed the button, the lightbulb turned on, and we indicated to them the specific time interval). Then, they were presented with 20 experimental trials (two for each delay and each condition), and they underwent a training session to familiarize themselves with the different temporal intervals. Once they learned to distinguish the different intervals efficiently (i.e., they got an accuracy of at least 80%), they proceeded with the testing phase.

### Statistical analyses—Power analysis

To calculate the sample size, we conducted a formal power analysis using the software G*Power (Faul et al., 2007) based on the data and the effect size reported by Cioffi et al. (2017). In particular, we simulated a repeated-measures analysis of variance (ANOVA), with within–between interactions, and we calculated that we would have needed a sample size of 26 in each group to reliably (with a probability greater than 0.95) detect an effect size of δ = 0.40, assuming a two-sided criterion for detection that allows for a maximum Type I error rate of α = 0.008 (in order to take into account for multiple comparisons post hoc corrections). We decided to recruit 30 participants per group to consider possible outliers.

### Statistical analyses—Explicit agency

Data collected were analyzed by using the software Jamovi (Version 2.3.21.0). Agency ratings were taken as a direct measure of the explicit *sense* o*f agency* (the greater the rating, the higher the *sense of agency*). These ratings represented the dependent variable of the model, while the factors “Group” (elderly/young), “Condition” (active/passive), and “Delay” (200/400/600 ms) were the independent variables. Given the non-parametric distribution of the data (Shapiro–Wilk test’s *W* < 0.9,* p* < .048), we performed a nonparametric one-way ANOVA (Kruskal–Wallis test) to explore between-group effects and a nonparametric repeated-measures ANOVA (Friedman test), followed by Durbin–Conover post hoc tests, to explore within-group effects. A visual inspection of our data through the box plots did not highlight any outliers; thus, 30 participants for each group were considered in the analysis.

### Statistical analyses—Implicit agency

Data collected were analyzed by using the software Jamovi (Version 2.3.21.0). In line with the description of the intentional binding phenomenon (Haggard et al., 2002), the “time compression” (TC)—namely, the difference between the real duration and the estimated duration of the action–outcome delay—was taken as an indirect measure of the *sense* o*f agency* (the greater the compression, the higher the implicit *sense of agency*). In order to take into account possible group differences related to a generally diminished precision in time perception associated with the aging process, we considered the TC values of the passive condition as a baseline: we subtracted each subject’s mean TC in the passive (baseline) conditions from the mean TC for the same event in the active conditions (in line with the analyses performed in the original work on intentional binding by Haggard et al., 2002). Negative values indicate a stronger TC in active conditions compared with passive ones (intentional binding phenomenon).

This “baseline corrected TC” measure represented the dependent variable of the model. At the same time, the between-groups factor “Group” (elderly/young) and the within-group factor “Delay” (200/400/600 ms) were the independent variables. Mean RTs were inserted as a covariate of no interest. Given the parametric distribution of the data (Shapiro–Wilk test’s *W* > 0.96, *p* > .05 for each considered variable), we tested this statistical model using a parametric mixed ANOVA. Significant interactions were explored using Bonferroni-corrected post hoc comparisons. Effect sizes were calculated using Cohen’s *d* starting. Based on a visual inspection of our data through the box plots, three participants were classified as outliers (two participants in the elderly group and one in the younger group). Thus, 28 participants for the elderly group and 29 for the younger group were considered in the analysis (in line with our power analysis estimates, see above). 

## Results

### Explicit agency

We observed significant group differences. Specifically, younger participants reported higher judgments of agency compared with the older subjects in the active condition, at 200 ms of delay between the action and the outcome, χ^2^(1) = 5.93, *p* = .015, ε^2^ = 0.10. This difference was not present in the other conditions/delays—passive 200 ms, χ^2^(1) = 1.82, *p* = .18, ε^2^ = 0.03; passive 400 ms: χ^2^(1) = 1.80, *p* = .18, ε^2^ = 0.03; passive 600 ms: χ^2^(1) = 0.75, *p* = .39, ε^2^ = 0.01; active 400 ms: χ^2^(1) = 1.97, *p* = .16, ε^2^ = 0.03; active 600 ms: χ^2^(1) = 0.5, *p* = .48, ε^2^ = 0.008.

We highlighted a significant difference between the active and passive trials. Judgments of agency were significantly higher for both groups in the active conditions when compared with the passive ones, for all the temporal delays. Young group—active 200 ms vs. passive 200 ms: Durbin–Conover test = 12.56, *p* < .001; active 400 ms vs. passive 400 ms: Durbin–Conover test = 10.67, *p* < .001; active 600 ms vs. passive 600 ms: Durbin–Conover test = 10.4, *p* < .001. Older group—active 200 ms vs. passive 200 ms: Durbin–Conover test = 7.76, *p* < .001; active 400 ms vs. passive 400 ms: Durbin–Conover test = 7.65, *p* < .001; active 600 ms vs. passive 600 ms: Durbin–Conover test = 7.86, *p* < .001.

Moreover, younger’s agency judgments were higher in the active conditions at 200 ms, when compared with longer time delays (200 ms vs. 400 ms: Durbin–Conover test = 1.96, *p* = .05; 200 ms vs. 600 ms: Durbin–Conover test = 3.11, *p* = .002; 400 ms vs. 600 ms: Durbin–Conover test = 1.15, *p* = .25. This temporal modulation was not present for the passive condition (200 ms vs. 400 ms: Durbin–Conover test = 0.07, *p* = .95; 200 ms vs. 600 ms: Durbin–Conover test = 0.94, *p* = .34; 400 ms vs. 600 ms: Durbin–Conover test = 0.88, *p* = .38.

**Fig. 2:**
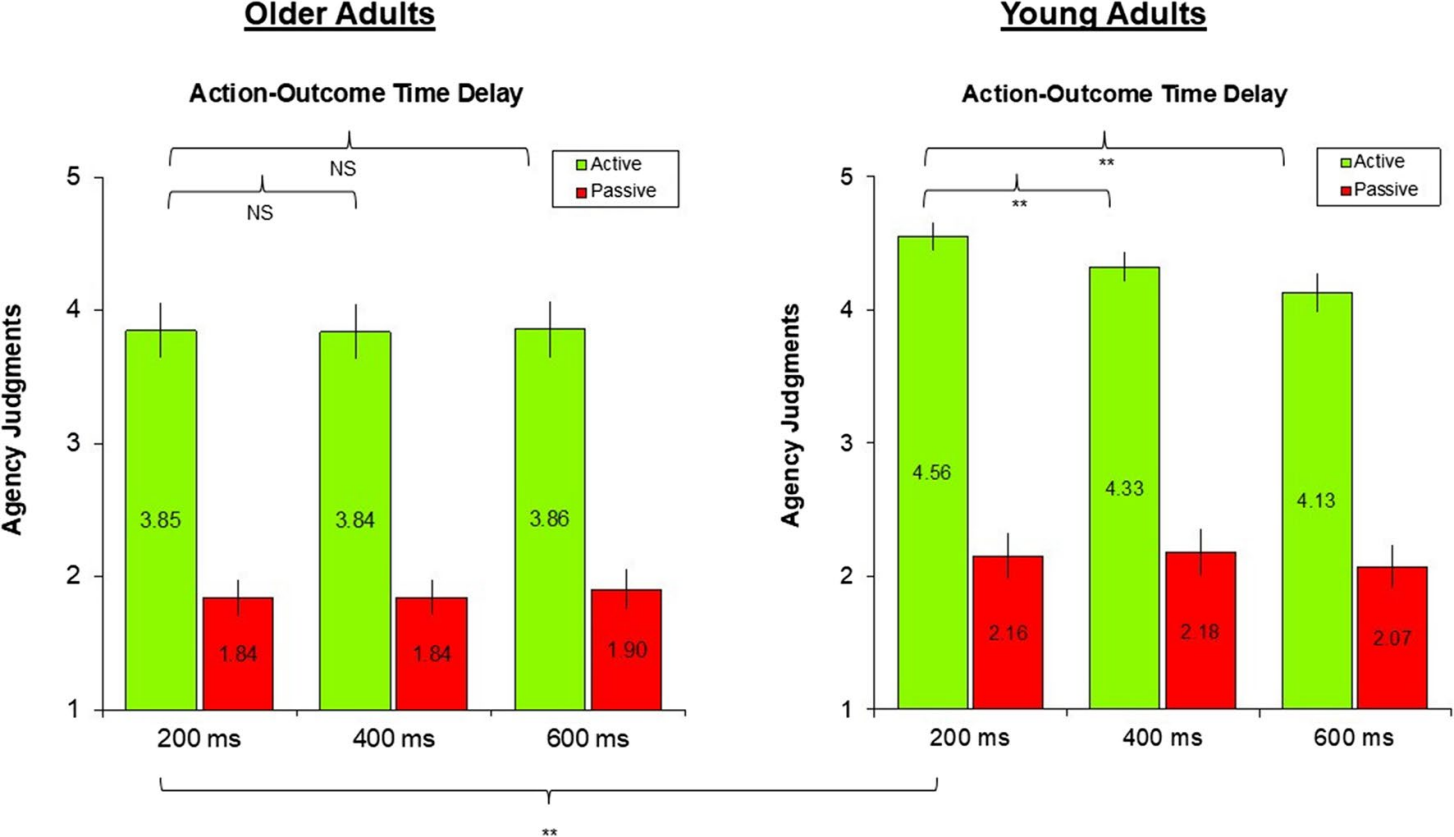
Mean and standard error values of agency judgments. Older participants showed reduced agency judgments when compared with the younger participants and did not exhibit a significant temporal modulation whereby explicit agency decreases as the temporal delay between the action and an outcome increases. (Color figure online)

Crucially, the older group did not show such temporal modulation in active nor in passive conditions—active: 200 ms vs. 400 ms: Durbin–Conover test = 0.26, *p* = .79; 200 ms vs. 600 ms: Durbin*-*Conover test = 0.1, *p* = .92; 400 ms vs. 600 ms: Durbin–Conover test = 0.37, *p* = .71; passive: 200 ms vs. 400 ms: Durbin–Conover test = 0.16, *p* = 0.87; 200 ms vs. 600 ms: Durbin–Conover test = 0.00, *p* = 1; 400 ms vs. 600 ms: Durbin–Conover test = 0.16, *p* = 0.87. See Fig. 2 and Table 1 for descriptive statistics.

**Table 1:** Descriptive statistics of explicit agency data (mean and standard deviation of judgments of agency) for each group (elderly/young participants), each condition (active/passive), and each temporal delay (200/400/600ms)

		Delay					
		Passive condition			Active condition		
	Group	200	400	600	200	400	600
Mean	Elderly	1.84	1.84	1.9	3.85	3.84	3.86
	Young	2.16	2.18	2.07	4.56	4.33	4.13
Standard deviation	Elderly	0.747	0.77	0.852	1.15	1.13	1.14
	Young	0.937	0.973	0.898	0.59	0.631	0.83

To sum up, older participants showed reduced agency judgments when compared with the younger participants and did not exhibit a significant temporal modulation whereby explicit agency decreases as the temporal delay between the action and an outcome increases.

### Implicit agency

We found the factor *delay* was not significant, *F*(2, 108) = 2.32, *p* = .10, ${\eta }_{{\text{partial}}}^{2}$ = 0.04, as the factor *group*, *F*(1, 54) = 0.96, *p* = .33, ${\eta }_{{\text{partial}}}^{2}$ = 0.02. However, we found a significant Group × Delay interaction, *F*(2, 108) = 7.93, *p* < .001, ${\eta }_{{\text{partial}}}^{2}$ = 0.13, and a non-significant Mean RTs × Delay, *F*(2, 108) = 0.60, *p* = .55, ${\eta }_{{\text{partial}}}^{2}$ = 0.01, interaction. The significant Group × Delay interaction was explored with post hoc between- and within-group comparisons.

### Between-group post hoc comparisons

We observed that the baseline corrected TC was significantly higher in young participants compared with the elderly subjects, only when the action–outcome delay was 200 ms between the movement and the lighting of the lightbulb (post hoc test young group vs. older group at 200 ms of delay between the movement and its consequence), *t*(54) = 3.08, mean difference = 68.99, *SE* = 22.4, corrected *p* = .018, Cohen’s *d* = 0.82; (post hoc test young group vs. older group at 400 ms of delay between the movement and its consequence), *t*(54) = 1.28, mean difference = 35.07, *SE* = 27.3, corrected *p* > .99, Cohen’s *d* = 0.34; (post hoc test young group vs. older group at 600 ms of delay between the movement and its consequence), *t*(54) = −1.81, mean difference = −48.99, *SE* = 27.1, corrected *p* = .46, Cohen’s *d* = −0.48.

### Within-group post hoc comparisons

Moreover, we observed that the baseline corrected TC was significantly higher in young participants when there was a temporal interval between the movement and the lighting of the light bulb was 200 ms, post hoc test 200 ms vs. 400 ms of delay between the movement and its consequence: *t*(54) = −2.92, mean difference = −61.87, *SE* = 21.2, corrected *p* = .015, Cohen’s *d* = −0.54; post hoc test 200 ms vs. 600 ms of delay between the movement and its consequence: *t*(54) = −6.09, mean difference = −145.78, *SE* = 23.9, corrected *p* < .001, Cohen’s *d* = −1.13.

**Fig. 3:**
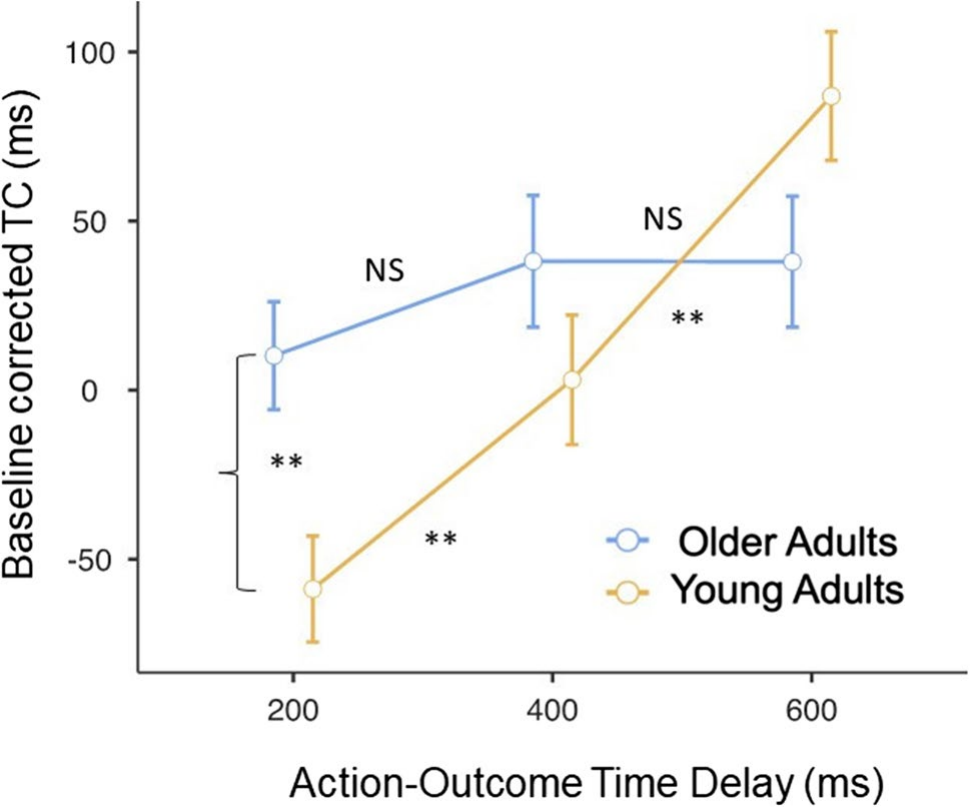
Mean and standard error of baseline corrected TC values. Baseline corrected TC values were significantly higher (i.e., more negative) in young participants when the action–outcome delay was 200 ms. This indicates a stronger intentional binding effect in young subjects compared with the older group. (Color figure online)

This temporal modulation was not present in the elderly subjects, post hoc test 200 ms vs. 400 ms of delay between the movement and its consequence: −(54) = −1.29, mean difference = −27.95, *SE* = 21.6, planned-corrected *p* = .60, Cohen’s *d* = −0.24; post hoc test 200 ms vs. 600 ms of delay between the movement and its consequence: t(54) = −1.14, mean difference = −27.8, *SE* = 24.4, planned-corrected corrected *p* = .77, Cohen’s *d* = −0.21. See Fig. 3 and Table 2 for descriptive statistics.

**Table 2:** Descriptive statistics of implicit agency data (mean and standard deviation of baseline-corrected time compression values) for each group (elderly/young participants) and each temporal delay (200/400/600ms)

		Delay		
	Group	200	400	600
Mean	Elderly	10.4	37.9	38.3
	Young	-59	3.34	86.7
Standard deviation	Elderly	95	109	98.3
	Young	71.6	96.1	105

To sum up, older participants did not show the expected intentional binding effect observed in younger subjects when the sensory outcome occurred 200 ms after the movement.

## Discussion

### Explicit agency

We observed that both groups reported higher agency judgments in active trials with respect to passive ones. This was true for all the time intervals between the active/passive pressing of the button and the lightening of the light bulb, suggesting a stronger agency experienced over the external effects that were actively caused by their voluntary movements.

However, when comparing the two groups, older adults exhibited lower agency judgments, indicating a decreased sense of agency perceived toward the effects of their voluntary actions, since the reduction was present only for active trials and not for passive ones. Moreover, group differences were found only when the time interval between the action and the sensory effects caused by such action was short. Indeed, we observed that young participants consistently reported significantly higher levels of agency when the action–outcome delay was 200 ms, while elderly participants did not show this temporal modulation.

These results confirm our previous findings whereby the sense of agency is stronger when the temporal delay between actions and their outcomes is 200 ms. Yet the comparison between the two groups revealed that the agency experience is significantly reduced in older adults, and it is not influenced by the time delay of the external outcome. This finding might indicate their lower reliance on external sensory stimuli when judging the agency experience due to the decreased sensitivity that characterizes the process of aging, and a more prominent commitment to internal motor signals (Boisgontier & Nougier, 2013; Lafargue et al., 2013), as already hypothesized by Metcalfe et al. (2010) and Cioffi et al. (2017) using two clearly different experimental tasks (i.e., the moving of a cursor on a screen under different manipulations and a vicarious agency paradigm).

Cioffi et al. (2017) further explored their findings, showing that older adults performed significantly better during proprioceptive and interoceptive tasks. Thus, having more precise proprioceptive information may lead older adults to increase the weight attributed to internal cues and diminish the reliance on external cues during the process of agency attribution. This may also explain why older adults were less susceptible to the vicarious agency illusion measured in their experiment. In the next paragraph, we will discuss whether this hypothesis can also apply to implicit agency measures collected in our experiment.

### Implicit agency

We observed a stronger temporal compression (indirect index of implicit agency) for younger subjects when the action–outcome delay was 200 ms, while older adults did not show any intentional binding effect. Crucially, this difference cannot be interpreted as the consequence of a generally diminished precision in time perception associated with the aging process since we considered the values of the passive condition as a baseline. Active and passive trials only differ for the absence or presence of motor intentionality, while the cognitive skills required to make the temporal judgments are the same in both conditions.

We can frame this evidence in the domain of the intentional binding experiments where the authors manipulated the reliability of the sensory external action outcomes, thus somehow simulating the condition of uncertainty and noise that we hypothesized characterizes our older participants’ sensory perceptions. Wolpe et al. (2013) showed a reduced intentional binding effect in the condition of high sensory uncertainty due to increased background noise (Wolpe et al., 2013). Based on these results, we can speculate that our elderly participants might perceive the external sensory outcomes of our experiment as noisier and thus less reliable. Consequently, their agency experience may rely more on internal representations, reducing the intentional binding effect.

This is also in line with a recent study by the same group: Wolpe et al. (2016) demonstrated that sensorimotor attenuation, a reduction in the perceived intensity of sensations from self-generated compared with external actions considered an implicit marker of agency, increased with age. The authors discussed this enhancement as the result of a “shift from noisier sensory information towards internal prediction associated with aging” (p. 8), similar to what was hypothesized for our results (Wolpe et al., 2016).

In the next paragraph, we will try to address these changes within the more general cognitive models of motor control.

### Cues from cognitive models of motor control and conclusions

Our results provide further support for the cue integration theory, whereby the sense of agency arises from a weighted integration of internal and external cues. Importantly, the relative influence of the different information sources may be linked to their reliability, with the more reliable source of information dominating the agentic experience (Moore et al., 2009; Synofzik et al., 2008).

The reduced sensitivity that characterizes the aging process may lead toward a lower reliance on sensory stimuli during the agency attribution process, and this would explain the group differences observed in our study. In particular, the general reduction of agency in aging may be explained by a sub-optimal and unbalanced integration of internal sensorimotor and external sensory cues, with an increased reliance on the former and lower confidence in the latter.

At first glance, an increased reliance on internal stimuli may seem advantageous as it may point to a more robust experience of agency in older adults. However, this is not what our results indicate. Yet the experience of agency in older adults is less sensitive to compelling external cues, suggesting that they may mainly focus on their actions without considering the external effects produced and overlooking potentially useful sources of information. As such, agency processing in older adults may be considered as not optimal.

Interestingly, a decreased sense of agency was also found in a group of 10-year-old children (Cavazzana et al., 2014). This evidence, combined with our findings demonstrating a general agency reduction in older adults, indicates that the sense of agency may follow a developmental trend: It may be gradually acquired during ontogenesis, reaching its maximum levels during adulthood and then decreasing with older age, following an inverted U-shaped trajectory, as hypothesized for other motor functions (Zapparoli et al., 2022).

In conclusion, our research represents a substantial contribution to the comprehension of agency mechanisms, corroborating the results from the literature on the ontogenetic development of the sense of agency. In future studies, it might be interesting to investigate the neural processes mediating such behavioral effects.

## Data Availability

The data is publicly accessible online **(**https://osf.io/gh8bk/).
